# Machine Learning Analysis of Type VII Secretion System Expression and Its Relationships With Virulence Traits and Antibiotic Resistance in Staphylococcus aureus

**DOI:** 10.7759/cureus.104870

**Published:** 2026-03-08

**Authors:** B Nirmala, Manju O Pai, Gaurav Badoni, Ganesh Kumar Verma, Ankur Kumar, Balram J Omar

**Affiliations:** 1 Microbiology, All India Institute of Medical Sciences, Rishikesh, Rishikesh, IND; 2 Molecular Biology, Proteomics and Metabolics, All India Institute of Medical Sciences, Rishikesh, Rishikesh, IND; 3 Biochemistry, All India Institute of Medical Sciences, Rishikesh, Rishikesh, IND; 4 Microbiology, Glocal University, Saharanpur, Saharanpur, IND

**Keywords:** antibiotic resistance, machine learning, staphylococcus aureus, type vii secretion system, virulence factors

## Abstract

Background

*Staphylococcus aureus (S. aureus)* remains a high-priority pathogen due to its extensive virulence arsenal, immune evasion strategies, and rising multidrug resistance, particularly in methicillin-resistant strains (MRSA). The type VII secretion system (T7SS), encoded by the ess locus, contributes to persistence, immune modulation, and interbacterial competition. Although recognized as a key virulence determinant, its regulatory interplay with other virulence traits and resistance mechanisms remains unclear. Understanding these relationships is essential to identifying novel antivirulence therapeutic targets.

Objectives

This study aimed to dissect the networks linking the T7SS to enzymatic virulence factors, antibiotic resistance, and environmental triggers using an integrated wet-lab and machine-learning approach.

Methods

We combined microbiological assays with machine-learning analyses to quantify associations between T7SS and the production of DNase, hemolysin, protease, lipase, and staphyloxanthin. Modulation of T7SS expression was assessed under physical (ultraviolet light), chemical (sodium hypochlorite disinfectant), and biological [coculture with *Escherichia coli (E. coli)]* conditions. A total of 150 clinical *S. aureus* isolates were evaluated for resistance profiles and phenotypic clustering.

Results

Among 150 clinical isolates, 74% were methicillin-resistant, 0.6% were vancomycin-resistant, and 88% were multidrug-resistant. T7SS expression showed positive correlations with virulence factors but no association with MRSA status or antibiotic resistance. Machine learning identified protease as the strongest predictor of T7SS expression and revealed clustering of high-expression phenotypes, highlighting complex interdependencies often overlooked by conventional approaches. Environmental factors significantly influenced T7SS expression: UV light downregulated it 1.5-fold, whereas sodium hypochlorite and *E. coli* coculture upregulated it by 2.5-fold and 2.2-fold, respectively. The positive correlation between T7SS and other virulence determinants suggests regulation via a shared accessory gene regulator (agr) system, though T7SS appears to function independently of resistance traits. Future studies should explore whether agr inhibitors can suppress T7SS and mitigate infection severity.

Discussion

The integrative analysis combining wet-lab microbiological assays with machine learning suggests that T7SS expression is associated with multiple virulence determinants in *S. aureus*. These findings indicate that T7SS may function as part of a broader virulence regulatory network rather than acting independently.

Conclusion

Overall, this study highlights the potential value of integrating experimental microbiology with computational approaches to explore complex virulence relationships in clinical *S. aureus* isolates. Further mechanistic and multi-center studies are required to validate these associations and clarify the regulatory pathways underlying T7SS activity.

## Introduction

Despite decades of research, *Staphylococcus aureus (S. aureus)* continues to surprise clinicians and scientists with its remarkable ability to exploit an arsenal of virulence factors to invade tissues [1], resist antibiotics [2], and evade the immune system [3-5]. This gram-positive pathogen, prioritised by the WHO for its public health impact [6], is notorious for causing infections that range from harmless skin lesions to life-threatening pneumonia and endocarditis [1]. The challenge is magnified by methicillin-resistant strains (MRSA), which compound virulence with multidrug resistance [7]. Among these, the type VII secretion system (T7SS) has emerged as a critical weapon [8], yet its connections to other virulence traits remain poorly understood.

The T7SS, also known as the ESAT-6-like secretion system (ESS), is encoded by the ess locus and comprises multiple transmembrane components and secreted effectors, including EsxA, which serves as a marker of T7SS activity [9-10]. T7SS contributes to infection persistence by lysing neutrophils [10], protecting against antimicrobial fatty acids [11], facilitating antibacterial competition [12], and modulating host immunity [13]. While the T7SS is a recognized virulence determinant, its relationships with other virulence factors and regulatory systems such as agr, SaeRS, and SarA remain incompletely understood [14-17].

To our knowledge, limited studies have examined the relationship between T7SS expression and other enzymatic virulence phenotypes in *S. aureus*, or evaluated how defined environmental stressors influence T7SS transcriptional responses. We hypothesized that esxA (T7SS marker) expression would be associated with the levels of key virulence factors (DNase, hemolysin, protease, lipase, and staphyloxanthin) and modulated by physical (UV irradiation), chemical (sodium hypochlorite), and biological (coculture with *E. coli*) exposures.

Because conventional statistical approaches may not fully capture multidimensional co-expression structure, we integrated wet-lab microbiological assays with exploratory machine-learning analyses to characterize patterns of association, clustering behavior, and feature contributions related to esxA expression. These computational methods were applied as data-structuring tools rather than as predictive clinical models.

Using this combined framework, we aimed to systematically evaluate associations between T7SS expression, virulence phenotypes, antibiotic resistance profiles, and environmental responsiveness in clinical *S. aureus* isolates.

This article was previously posted to the Research Square preprint server on August 25, 2025 [18].

## Materials and methods

Bacterial collection, identification, and antibiotic susceptibility testing

This study was conducted in a tertiary care setting and included 150 clinical *S. aureus* isolates collected at AIIMS Rishikesh between September 2023 and January 2025 from diverse specimens (pus, blood, tissue, sputum, urine, and body fluids). Control strains comprised *S. aureus* American Type Culture Collection (ATCC) 25923, *E. coli* ATCC 25922, and the T7SS-positive *S. aureus* RN6390 (New York University School of Medicine). Isolates were identified using blood agar culture, standard biochemical tests, the VITEK® 2 Compact system (bioMérieux, Marcy-l’Étoile, France), and MALDI-TOF (Bruker Biotyper, Bremen, Germany), then preserved in glycerol stocks at -20°C and -80°C and maintained on nutrient agar at 4°C.

Methicillin resistance was determined by cefoxitin disc diffusion (30 µg) on Mueller-Hinton agar following Clinical and Laboratory Standards Institute (CLSI) 2019 criteria, with the USA 600 MRSA strain as a positive control. Antibiotic susceptibility was assessed using the VITEK® 2 Compact system with antimicrobial susceptibility testing (AST) p628 cards. The Institutional Ethics Committee approved the study (Letter No: AIIMS/IEC/23/294).

Detection and quantification of the T7SS in clinical isolates of *S. aureus*

Genomic DNA and total RNA were extracted from 24-hour blood agar cultures of *S. aureus* using HiPurA purification kits (HIMEDIA, Mumbai, India). Purity was assessed by agarose gel electrophoresis and the Tecan NanoQuant Plate™, used with the Infinite® 200 Pro microplate reader (Tecan Life Sciences, Männedorf, Switzerland). Polymerase chain reaction (PCR) amplification of the esxA gene was performed in 25 μl reactions containing Emerald dAMP GT Master Mix (Gene to Protein Pvt. Ltd., New Delhi, India) and specific primers (Table 1) using an Applied Biosystems Veriti™ 96-Well Thermal Cycler (Thermo Fisher Scientific, Waltham, USA). *S. aureus* 16S rRNA served as the positive control, and *E. coli* ATCC 25922 as the negative control. The primers utilized in this study were designed using the National Center for Biotechnology Information Basic Local Alignment Search Tool (NCBI BLAST) [19]. Their efficiency was validated with an in silico PCR amplification tool [20].

**Table 1:** Primer sequences utilized in this study. PCR: polymerase chain reaction; qPCR: quantitative polymerase chain reaction.

Gene	Primer Sequence (5′-3′)	Product Length (bp)	Purpose
esxA	GCAATGATTAAGATGAGTCCAGAGG TTATTGCAAACCGAAATTATTAGAA	291	PCR
esxA	TTACGGGCAAGGTTCAGACC ACAGCGTCAGCAGTGCTATT	198	qPCR
16 srRNA	ACGGTCTTGCTGTCACTTATA TACACATATGTTCTTCCCTAATAA	257	Internal control

For quantitative polymerase chain reaction (qPCR), RNA was reverse-transcribed to cDNA with G-Biosciences reagents. Reactions (20 μl) containing SYBR™ Green qPCR Master Mix (G-Biosciences, St. Louis, USA) were run on a C1000 Touch™ Thermal Cycler with CFX96™ Real-Time PCR Detection System (Bio-Rad Laboratories, Hercules, CA, USA) under standard cycling conditions. Amplicon specificity for both DNA and RNA analyses was confirmed by gel electrophoresis. Full protocols and thermal profiles are available in Appendices 1 and 2.

Detection of *S. aureus* virulence factors

Colonies from 24-hour blood agar cultures were suspended in 0.9% saline (0.5 McFarland standard). DNase activity was tested on DNase agar with methyl green (HIMEDIA Laboratories Pvt. Ltd., Mumbai, India); hemolysin on sheep blood agar (bioMérieux, Marcy-l’Étoile, France); protease on milk agar (HIMEDIA Laboratories Pvt. Ltd., Mumbai, India); and lipase on egg yolk agar (10% egg yolk in nutrient agar). Plates were incubated at 37°C for 24 hours. DNase and lipase were indicated by clearance zones, hemolysin by lysis, and protease by precipitation. Zone sizes categorized production as weak (≤6 mm), moderate (7-8 mm), or high (≥9 mm). Staphyloxanthin was assessed on nutrient agar based on pigment intensity. Capsule detection was performed using Maneval’s stain as previously described [21] with visualization under oil-immersion microscopy.

Correlations between the T7SS and other virulence factors of *S. aureus*

Sample size (n=84) was calculated in G*Power 3.1.9.7 (Heinrich Heine University Düsseldorf, Düsseldorf, Germany) to achieve 80% power with 95% confidence. The T7SS Ct values were correlated with virulence factor zone sizes by bivariate analysis in SPSS v21.0 (IBM Corporation, Armonk, NY) (p < 0.05); as lower Ct indicates higher expression, negative correlations reflect positive associations. Furthermore, T7SS expression was correlated with MRSA and drug resistance patterns.

Machine learning, dimensionality reduction, and resistance network analyses

All data were normalized using a standard scaler before analysis. Machine learning analyses were conducted in Python v3.9 (Python Software Foundation, Wilmington, USA). For dimensionality reduction and visualization, principal component analysis (PCA) was applied to capture variance in virulence factor expression, supported by scree plots and feature distributions to identify clustering and co-regulation patterns. Clustering analysis employed Balanced Iterative Reducing and Clustering using Hierarchies (BIRCH) pairplot visualization and K-means clustering to explore whether elevated EsxA expression correlated with other virulence factors.

Random Forest and SHapley Additive exPlanations (SHAP) analysis were conducted to quantify feature importance and rank contributions to EsxA expression, while Ridge regression examined the distribution of virulence factor levels. Interaction mapping involved constructing chord diagrams and line graphs to assess co-regulation among factors. A Markov Chain Model was developed to analyse transition dynamics based on feature co-occurrence.

For the antibiotic susceptibility data of 150 *S. aureus* isolates, preprocessing and normalization were performed to ensure consistency across variables. Heatmaps were used to visualize patterns of resistance, intermediate susceptibility, and sensitivity. Scatterplot clustering was applied to identify distinct resistance profiles. Violin plots were created to examine the distribution of resistance levels across antibiotics and assess the presence of mixed or bimodal resistance patterns. Chord diagrams were employed to map interactions and co-occurrence relationships among antibiotic resistance traits. A co-resistance network was generated in Gephi open-source software [University of Technology of Compiègne (UTC), Compiègne, France) with nodes representing antibiotics and edges denoting significant associations (p<0.05). Dark grey edges indicated strong co-resistance. Finally, a Markov transition model, using maximum likelihood estimation, evaluated the probabilities of resistance evolution between antibiotics.

All computational scripts used for preprocessing, clustering, machine-learning analyses, and network modeling are publicly available on GitHub (https://github.com/Nirmala-1997/Staphylococcus-ML-Virulence-AST) to support full reproducibility of the analyses.

Regulation of the T7SS by environmental factors

For assessment of physical stress, *S. aureus *colonies were cultured on blood agar and incubated overnight at 37°C. Plates were then exposed to ultraviolet (UV) irradiation using a 15-watt 254 nm UV lamp for 0, 10, 30, 60, or 120 minutes. For chemical treatment, bacterial suspensions adjusted to the 0.5 McFarland standard in 0.9% normal saline were treated with 0.1% sodium hypochlorite and incubated at 37°C for 60 minutes. For biological stress, *S. aureus* suspensions (0.5 McFarland) were cocultured with an equal volume of *E. coli *suspension (0.5 McFarland) for 60 minutes at 37°C.

Following each treatment, RNA was extracted, converted to cDNA, and analysed by quantitative PCR (qPCR) to quantify T7SS expression, using the 16S rRNA gene as an internal control. Untreated or monoculture suspensions served as respective controls. Changes in T7SS expression were calculated using the delta-delta Ct (2^-ΔΔCt) method. All experiments were performed in triplicate for reproducibility.

Statistical analysis

Graphs were created in Power BI, and diagrams in Canva (Canva Pty Ltd, New South Wales, Australia). The machine learning analyses were performed using Python v3.9. The scatterplot and statistical analyses, including the bivariate correlation coefficient test and paired t-test, were performed using SPSS software version 21.0, with statistical significance set at p < 0.05. The sample size power analysis was performed using G*Power 3.1.9.7.

## Results

Detection of capsule production

**Figure 1:**
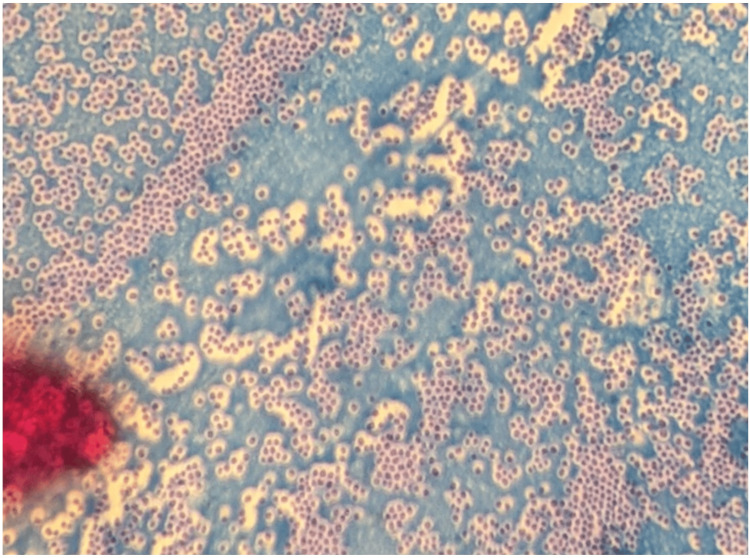
Capsule visualization in S. aureus using Maneval’s stain. Unstained capsules appear as clear halos surrounding magenta-red bacterial cells against a blue background.

Capsules were identified in all 150 clinical isolates of *S. aureus*. The background was dark blue, featuring unstained capsules alongside magenta-red bacteria (Figure 1).

Molecular detection and quantification of the T7SS in *S. aureus* clinical isolates

**Figure 2:**
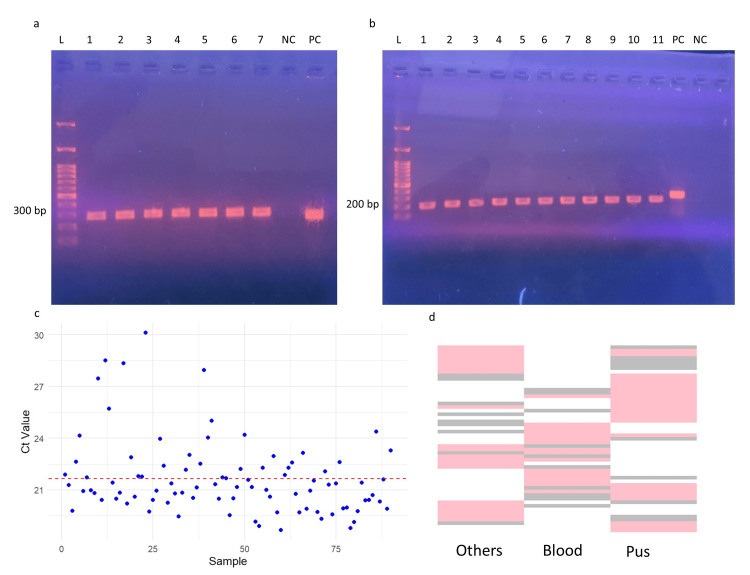
PCR and qPCR validation of esxA expression in S. aureus isolates. (a) PCR results: L – 100 bp ladder; lanes 1–7 – samples showing a positive 291 bp amplified PCR product; NC – negative control (ATCC *E. coli* 25922); PC – positive control (*S. aureus* 16S rRNA gene, 257 bp). (b) qPCR results: L – 100 bp ladder; lanes 1–11 – samples displaying a positive 196 bp amplified qPCR product; PC – positive control (*S. aureus* 16S rRNA gene, 257 bp); NC – no-template control. (c) Scatterplot showing esxA gene expression across samples, with Ct values ranging from 18 to 30. (d) Grouping of isolates by sample type demonstrating uniform T7SS expression across categories. Analysis was performed using Python v3.9.  PCR: polymerase chain reaction; qPCR: quantitative polymerase chain reaction.

Agarose gel electrophoresis revealed the presence of the PCR-amplified esxA gene product (291 bp). Among the 150 clinical isolates, all tested positive for the esxA gene, indicating that all the *S. aureus* strains contained the T7SS protein complex, while the negative control, the ATCC *E. coli* 25922 strain, displayed no band (Figure 2a). Subsequent RT-qPCR analysis quantified esxA expression across the clinical isolates, confirming its presence in all the samples. The amplified qPCR products were verified using agarose gel electrophoresis, which yielded a band of 198 bp (Figure 2b). However, the expression levels of these genes varied among the clinical isolates, with Ct values ranging from 18 to 30 (Figure 2c). The isolates were grouped by sample type, and the overall expression patterns were analysed using a heatmap (Figure 2d), which revealed a uniform T7SS expression across the samples, with minimal significant differences between categories.

Secretion of virulence factors

**Figure 3:**
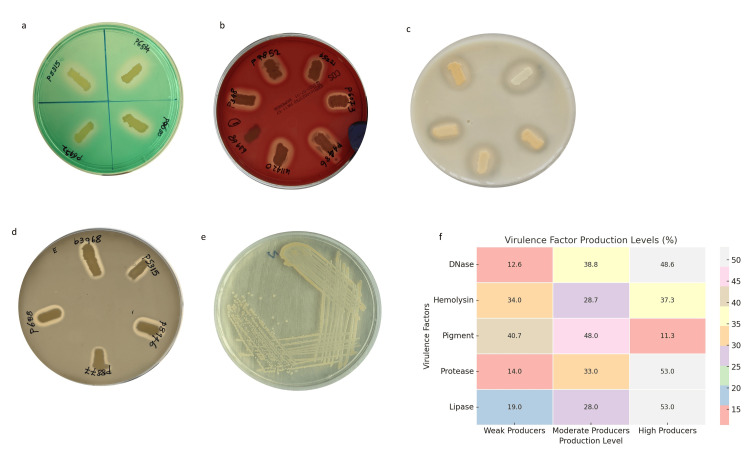
Representative images illustrating virulence factor production in clinical S. aureus isolates are shown. Panel (a) displays DNase activity on DNase agar supplemented with methyl green. Panel (b) shows hemolysin production evidenced by clear zones of hemolysis on sheep blood agar. Panel (c) demonstrates protease activity on milk agar through casein hydrolysis. Panel (d) depicts lipase production on egg-yolk agar. Panel (e) shows staphyloxanthin pigment production on nutrient agar. Panel (f) presents a heatmap summarizing the overall distribution of virulence factor expression levels among the *S. aureus* clinical isolates, categorized as weak, moderate, and high producers. Analysis was performed using Python v3.9.

Among the clinical *S. aureus* isolates, DNase activity was the most prominently expressed, with a majority of strains producing large clearance zones. In contrast, staphyloxanthin pigment production was comparatively low across isolates. Lipase and protease exhibited similar levels of expression, typically falling into moderate production categories (Figure 3).

Correlation of the T7SS and virulence factors

T7SS expression showed significant positive correlations with DNase (r=-0.314, p<0.01), hemolysin (r=-0.423, p<0.01), protease (r=-0.291, p<0.05), lipase (r=-0.339, p<0.05), and staphyloxanthin production (r=-0.188, p<0.05). A negative sign indicates that as the cycle threshold for T7SS expression decreases, the levels of virulence factors increase. This finding suggested that a common regulatory mechanism governs the T7SS and other virulence factors. However, no correlation was found between MRSA and the T7SS (r=0.039, p=0.71) or between the drug resistance pattern of *S. aureus* and the T7SS (r=0.012, p=0.91), indicating that these factors operate independently.

Machine learning analysis of virulence factor expression

**Figure 4:**
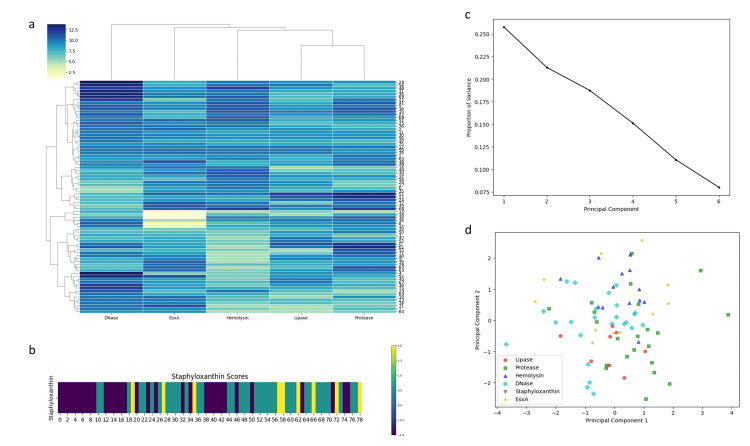
Comprehensive analysis of virulence factor expression in S. aureus isolates. (a) Heatmap depicting the expression levels of DNase, EsxA, hemolysin, lipase, and protease across clinical isolates, with hierarchical clustering of both samples and factors. (b) Distribution of staphyloxanthin production scores among the isolates. (c) Scree plot showing the proportion of variance explained by each principal component derived from virulence factor data. (d) Principal component analysis (PCA) scatterplot illustrating clustering patterns of isolates based on virulence factor expression. Analysis was performed using Python v3.9.

The expression profiles of virulence factors were first visualized in a heatmap (Figures 4a, 4b), showing that DNase was most abundantly expressed, while lipase and protease clustered closely due to similar expression patterns. PCA captured key variance (PC1: 25%, PC2: 22%), indicating that EsxA expression was distributed among other factors rather than forming discrete clusters (Figures 4c, 4d).

**Figure 5:**
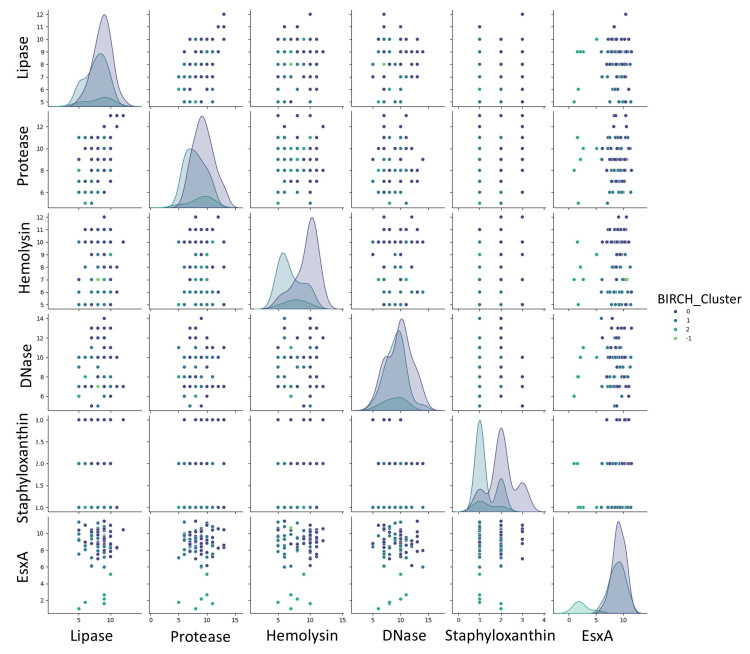
Pairwise analyses: scatterplot matrix displaying the distributions and pairwise relationships among lipase, protease, hemolysin, DNase, staphyloxanthin, and EsxA expression levels. Analysis was performed using Python v3.9.

To further explore these patterns, BIRCH clustering and pairwise scatterplots were used (Figure 5), revealing consistent associations between EsxA and other virulence determinants.

**Figure 6:**
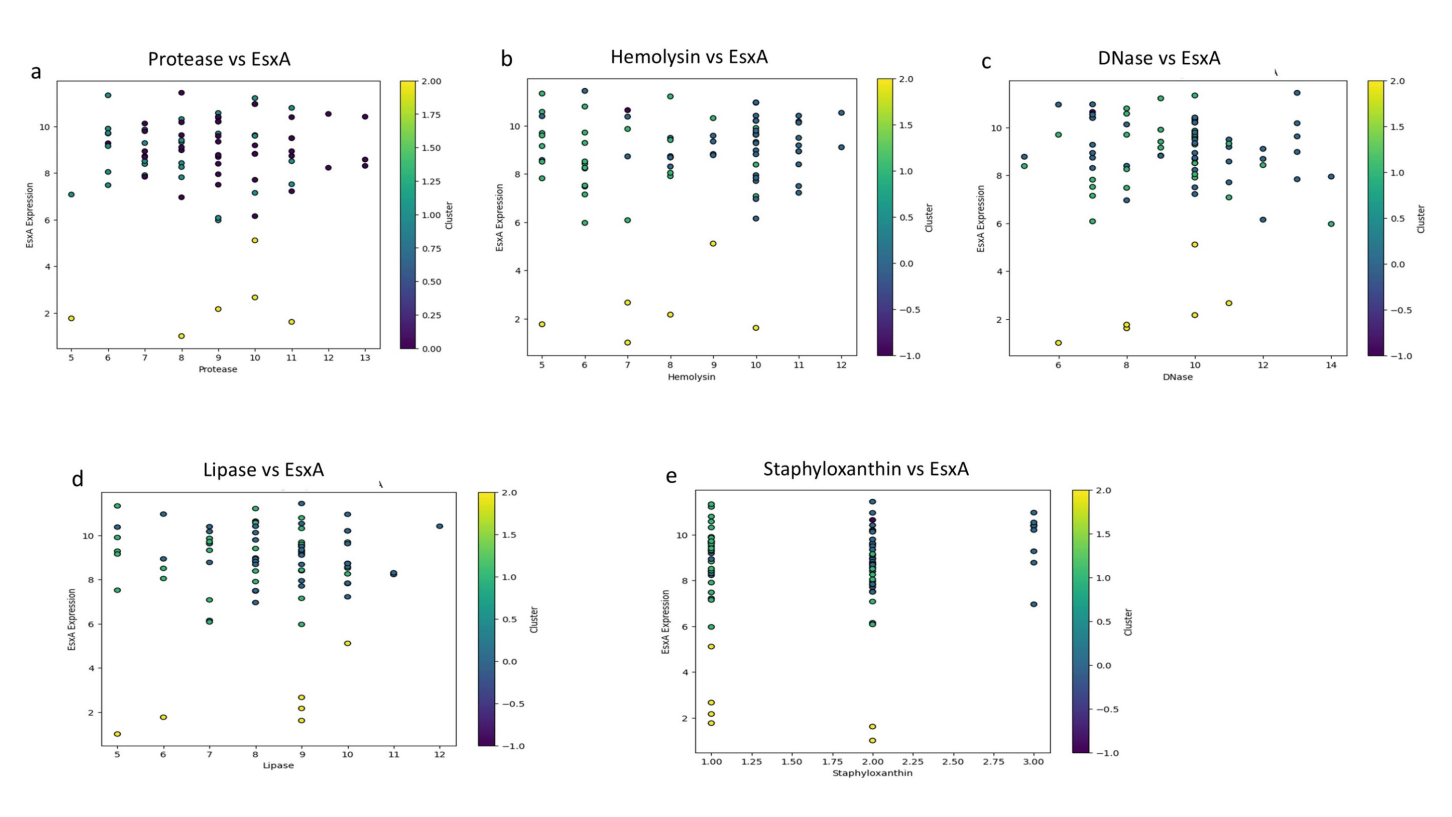
K-means clustering analyses of EsxA expression with individual virulence factors: protease (a), hemolysin (b), DNase (c), lipase (d), and staphyloxanthin (e). Data points are colored by cluster membership, highlighting patterns of co-expression and clustering among the different virulence determinants. Analysis was performed using Python v3.9.

K-means clustering of EsxA with individual factors confirmed that higher EsxA expression frequently coincided with elevated levels of protease, hemolysin, DNase, lipase, and staphyloxanthin (Figure 6).

**Figure 7:**
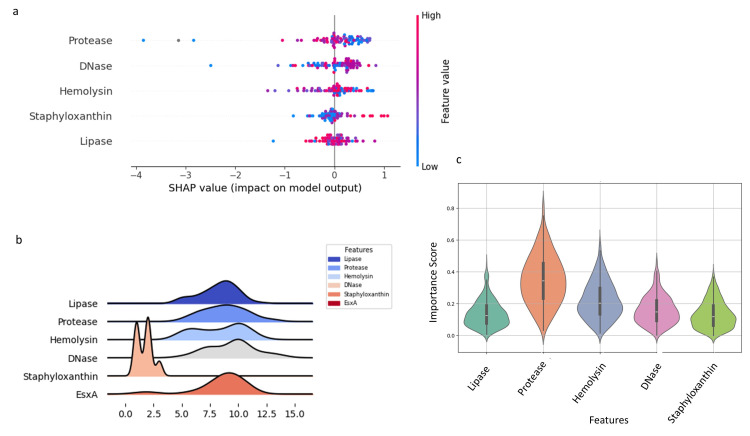
Machine learning-based assessment of virulence factor importance and expression distributions in S. aureus isolates. (a) SHAP value summary plot indicating the impact of individual virulence factors on model output, with color reflecting feature value magnitude. (b) Ridge plots illustrating the distribution patterns of lipase, protease, hemolysin, DNase, staphyloxanthin, and EsxA expression. (c) Violin plots of feature importance scores derived from random forest analysis, highlighting protease as the most influential predictor. Analysis was performed using Python v3.9.

Feature selection using SHAP analysis identified protease as the most influential predictor of overall expression patterns (Figure 7a). Ridge and violin plots showed unimodal distributions for lipase and protease, while DNase and hemolysin exhibited bimodal patterns (Figure 7b, 7c).

**Figure 8:**
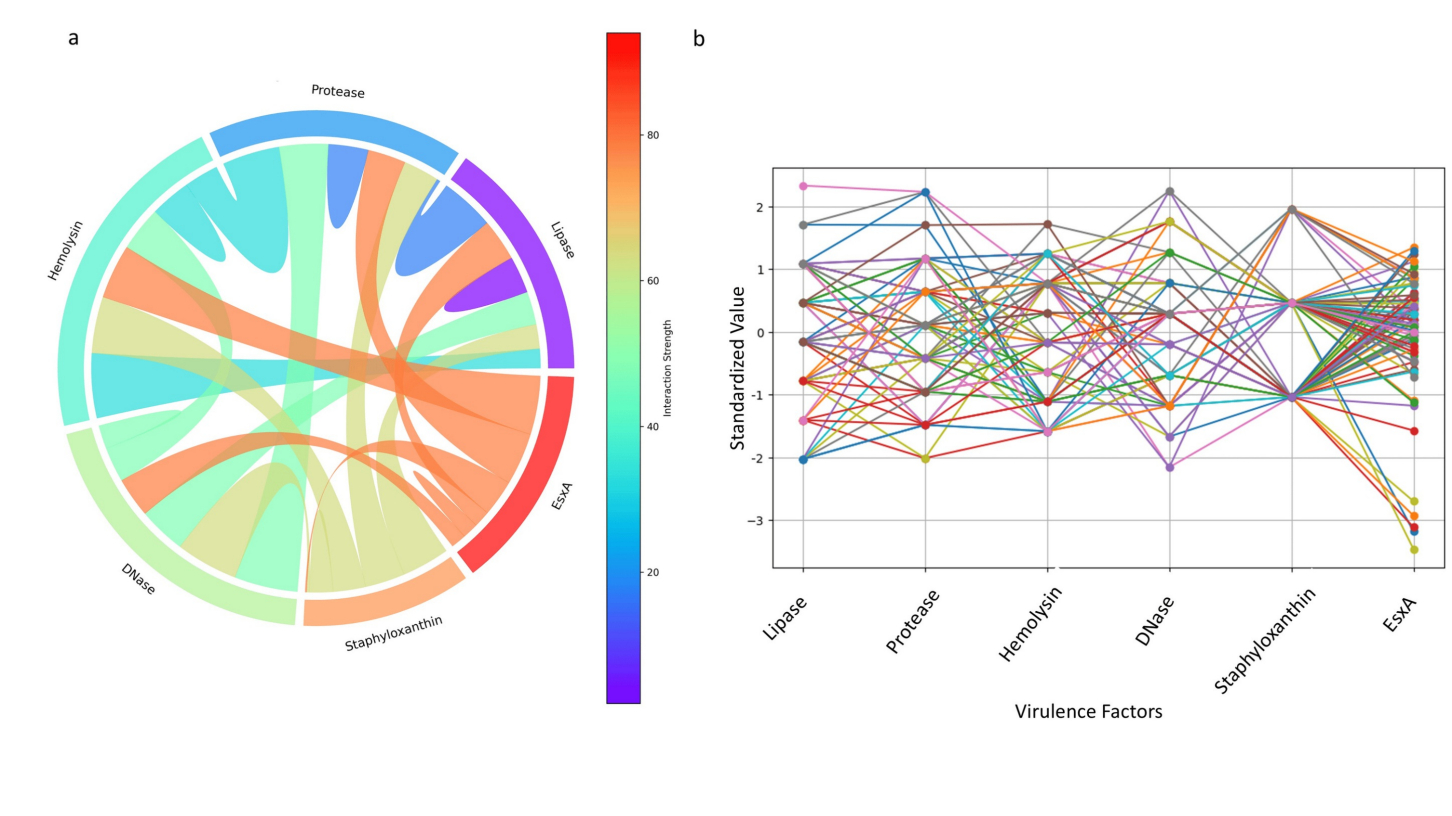
Visualization of interactions among virulence factors in S. aureus isolates. (a) Chord diagram illustrating the strength and direction of pairwise interactions between virulence factors. Colors indicate interaction strength (from low in purple to high in red), while the thickness of each chord reflects the magnitude of the association. (b) Line graph showing standardized expression values across all virulence factors, highlighting patterns of co-variation among isolates. Analysis was performed using Python v3.9.

To assess regulatory interactions, chord diagrams were constructed (Figure 8a), highlighting strong associations between EsxA and hemolysin. This was further supported by line graph analysis (Figure 8b), indicating co-variation in their expression, and an inverse relationship between staphyloxanthin and DNase.

**Figure 9:**
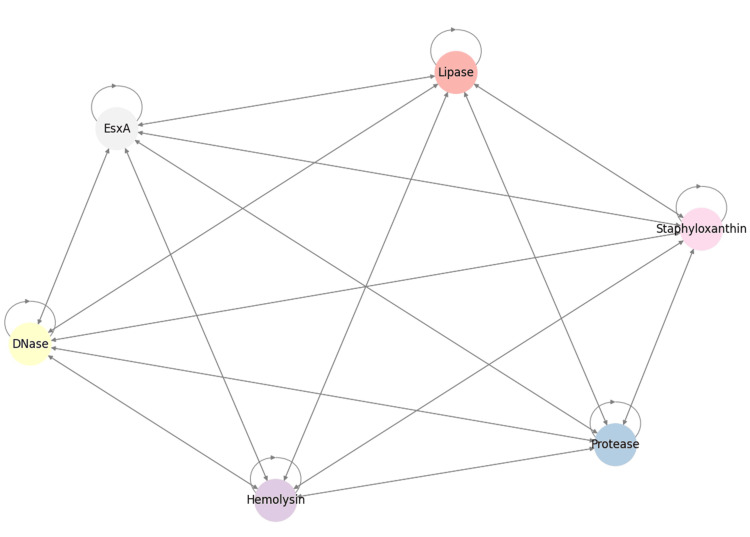
Markov chain model depicting probabilistic transitions and interconnections among virulence factors, suggesting potential regulatory relationships. Analysis was performed using Python v3.9.

Finally, a Markov Chain Model illustrated dynamic transitions among virulence factors (Figure 9). Lipase and DNase emerged as key hubs, with EsxA showing strong connectivity to DNase, lipase, and staphyloxanthin, highlighting its potential role in coordinating secretion-associated virulence.

Antibiotic susceptibility and resistance network analysis

Among the 150 clinical isolates, 74% (n=111) were identified as MRSA. Furthermore, 97.4% of the isolates were classified as vancomycin-sensitive *S. aureus* (VSSA), 2% were classified as vancomycin-intermediate *S. aureus* (VISA), and 0.6% were classified as vancomycin-resistant *S. aureus* (VRSA). Multidrug resistance (MDR) was detected in 88% of our isolates. Notably, we detected 0% extreme drug-resistant (XDR) *S. aureus*.

**Figure 10:**
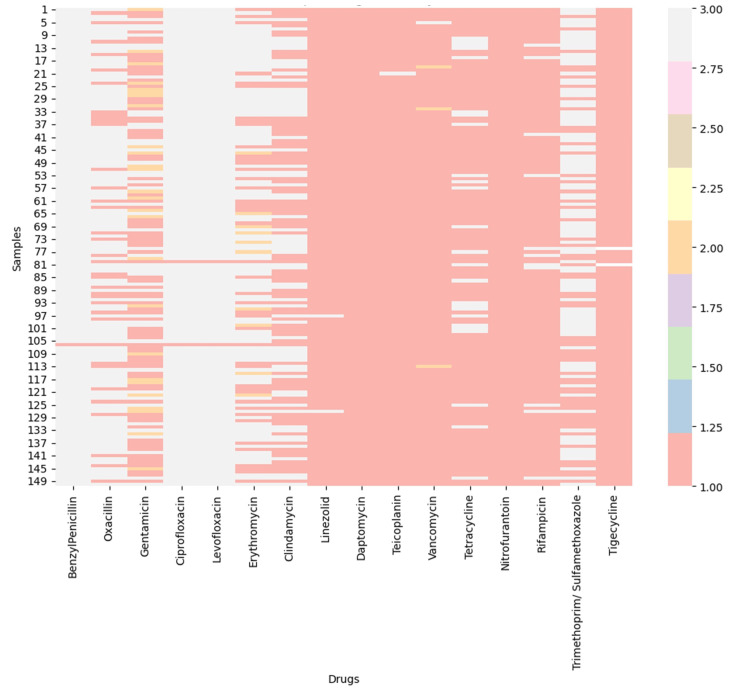
Heatmap showing resistance, intermediate susceptibility, and sensitivity profiles for each antibiotic, with red shades indicating higher sensitivity and lighter colors representing resistance. Analysis was performed using Python v3.9.

**Figure 11:**
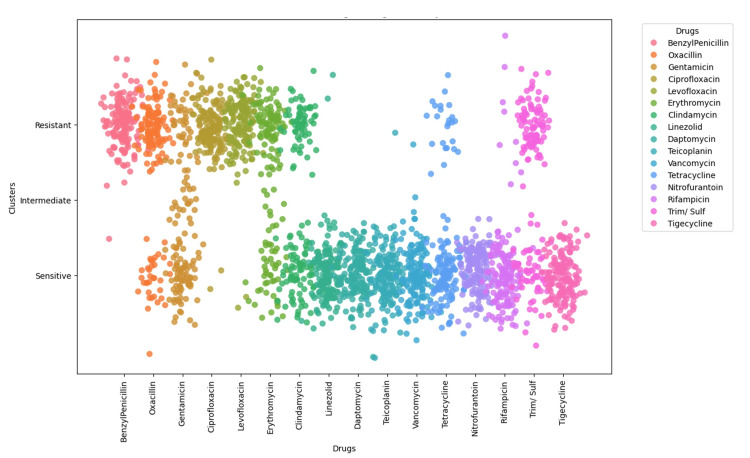
Visualization of antimicrobial susceptibility patterns across StaphylococScatterplot illustrating clustering of resistance patterns by drug, categorized into resistant, intermediate, and sensitive groups. Analysis was performed using Python v3.9.

**Figure 12:**
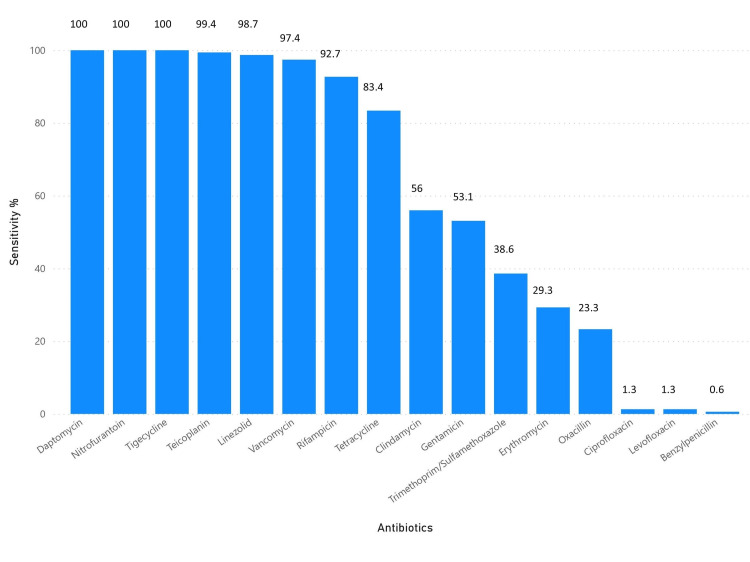
Bar plot displaying the percentage of isolates sensitive to each antibiotic. Graph was generated using Microsoft Power BI.

The overall resistance and sensitivity patterns are shown in Figures 10-12. Notably, no isolates were resistant to daptomycin, nitrofurantoin, or tigecycline, while nearly all were resistant to ciprofloxacin, levofloxacin, and benzylpenicillin.

**Figure 13:**
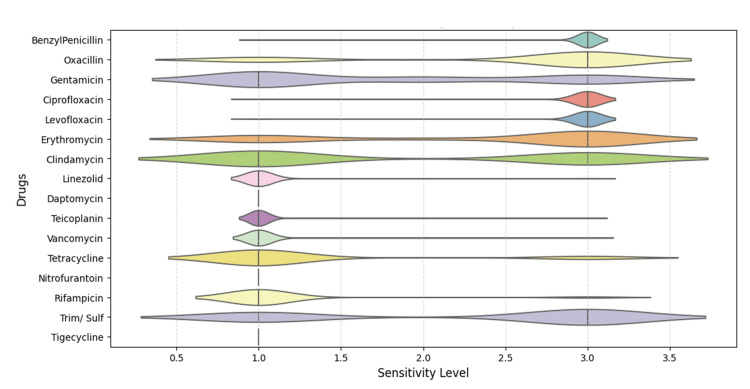
Violin plots depicting the distribution of sensitivity levels for each antibiotic, highlighting variability and multimodal patterns in susceptibility. Analysis was performed using Python v3.9.

Violin plots revealed bimodal distributions for trimethoprim-sulfamethoxazole, oxacillin, erythromycin, and clindamycin, while linezolid, daptomycin, tigecycline, and teicoplanin displayed narrow distributions indicative of high susceptibility (Figure 13).

**Figure 14:**
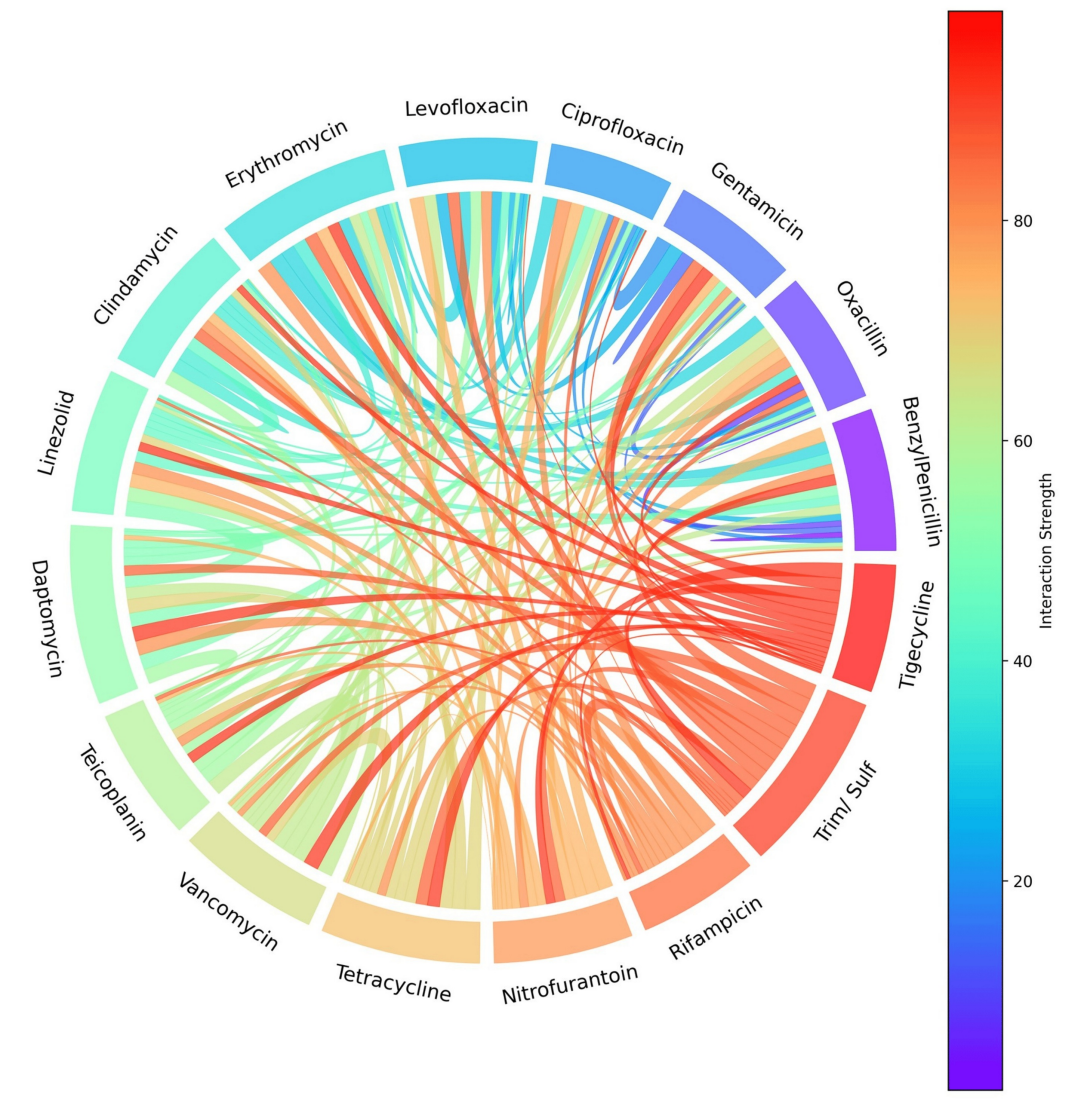
Chord diagram showing pairwise interaction strengths between antibiotics, with color gradients indicating the magnitude of association (from low in purple to high in red) and thicker chords representing stronger correlations in susceptibility or resistance profiles. Analysis was performed using Python v3.9.

To explore inter-drug relationships, chord analysis identified strong associations among tigecycline, trimethoprim-sulfamethoxazole, and rifampicin, and moderate interactions for vancomycin, teicoplanin, daptomycin, and linezolid. In contrast, beta-lactams and fluoroquinolones exhibited weaker pairwise correlations, reflecting more diverse resistance mechanisms (Figure 14).

**Figure 15:**
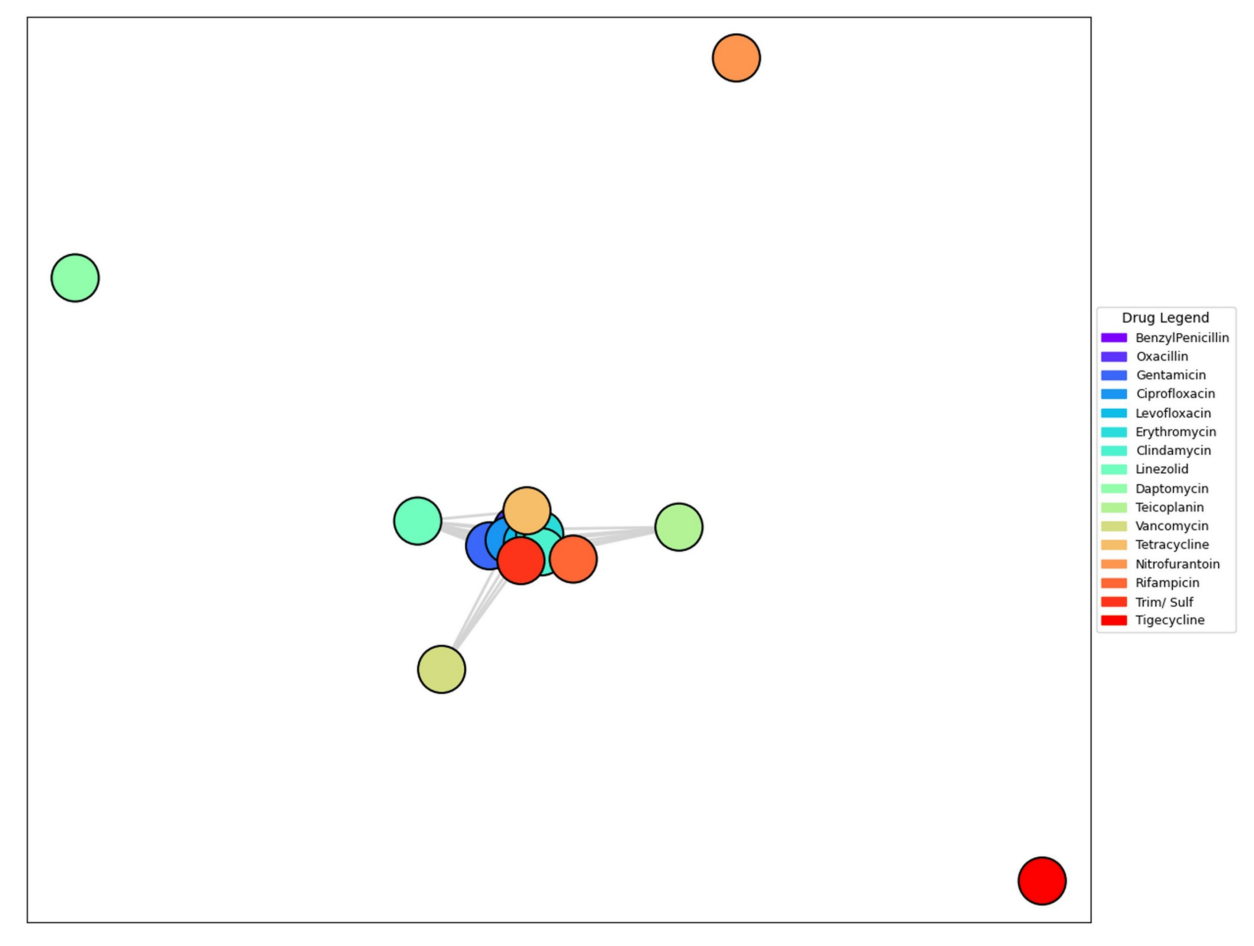
Co-resistive network graph displaying the multivariate relationships among antibiotics. Nodes are colored according to the drug legend, and their proximity indicates similarity in resistance patterns. Analysis was performed using Python v3.9.

Co-resistance network visualization highlighted a central cluster including gentamicin, fluoroquinolones, erythromycin, clindamycin, and rifampicin (Figure 15). Peripheral nodes such as tigecycline and nitrofurantoin appeared isolated.

**Figure 16:**
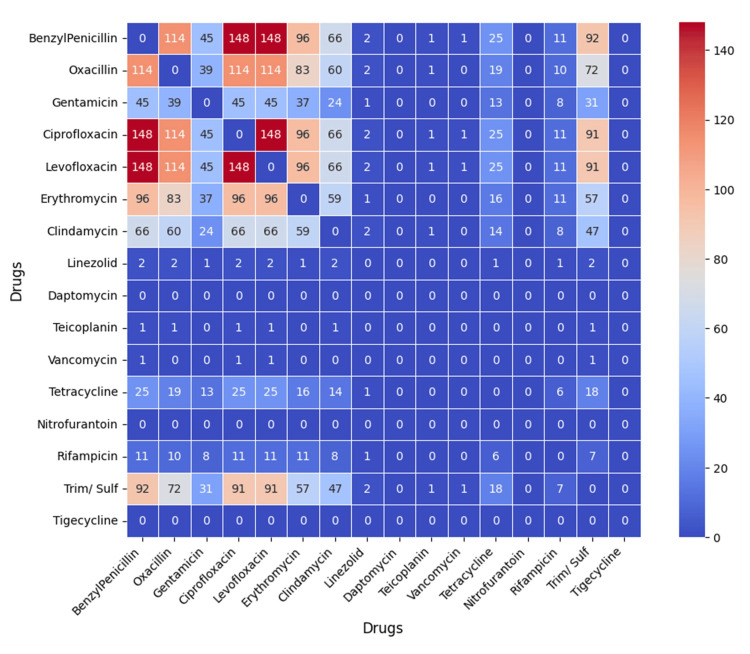
Co-occurrence heatmap showing the frequency of simultaneous resistance between antibiotic pairs, with higher counts indicated by darker red shading. Analysis was performed using Python v3.9.

The co-occurrence heatmap (Figure 16) showed frequent simultaneous resistance between ciprofloxacin and levofloxacin, with high co-resistance also involving benzylpenicillin and oxacillin. Gentamicin, clindamycin, and trimethoprim-sulfamethoxazole exhibited moderate co-occurrence, while linezolid, daptomycin, and tigecycline showed minimal overlap.

**Figure 17:**
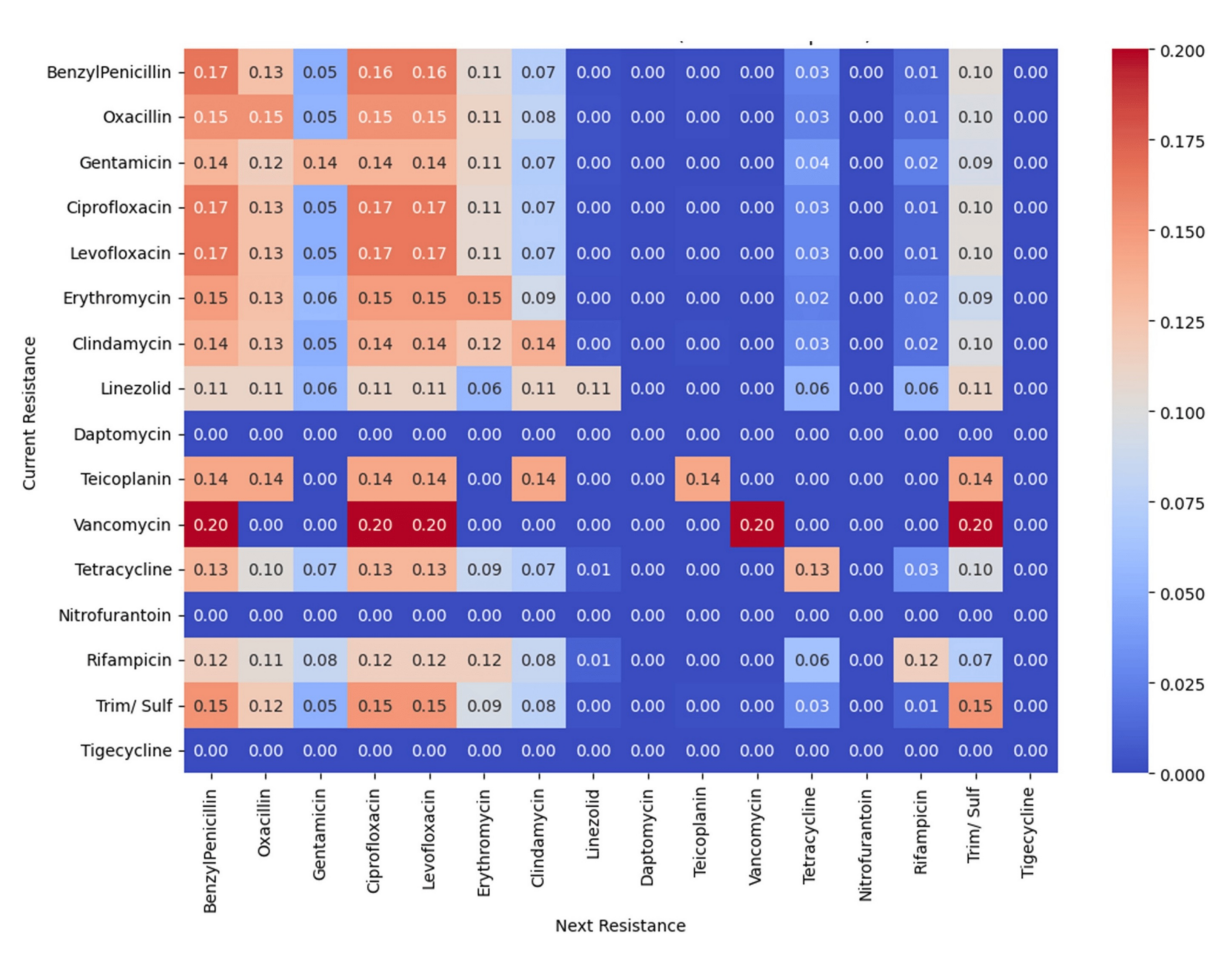
Markov transition matrix heatmap illustrating the probability of transitioning from resistance to one antibiotic (rows) to resistance to another (columns). Analysis was performed using Python v3.9.

The Markov transition matrix (Figure 17) indicated that vancomycin, fluoroquinolones, and benzylpenicillin had the highest probabilities of transitioning to broader resistance, whereas linezolid and tigecycline maintained low transition rates.

**Figure 18:**
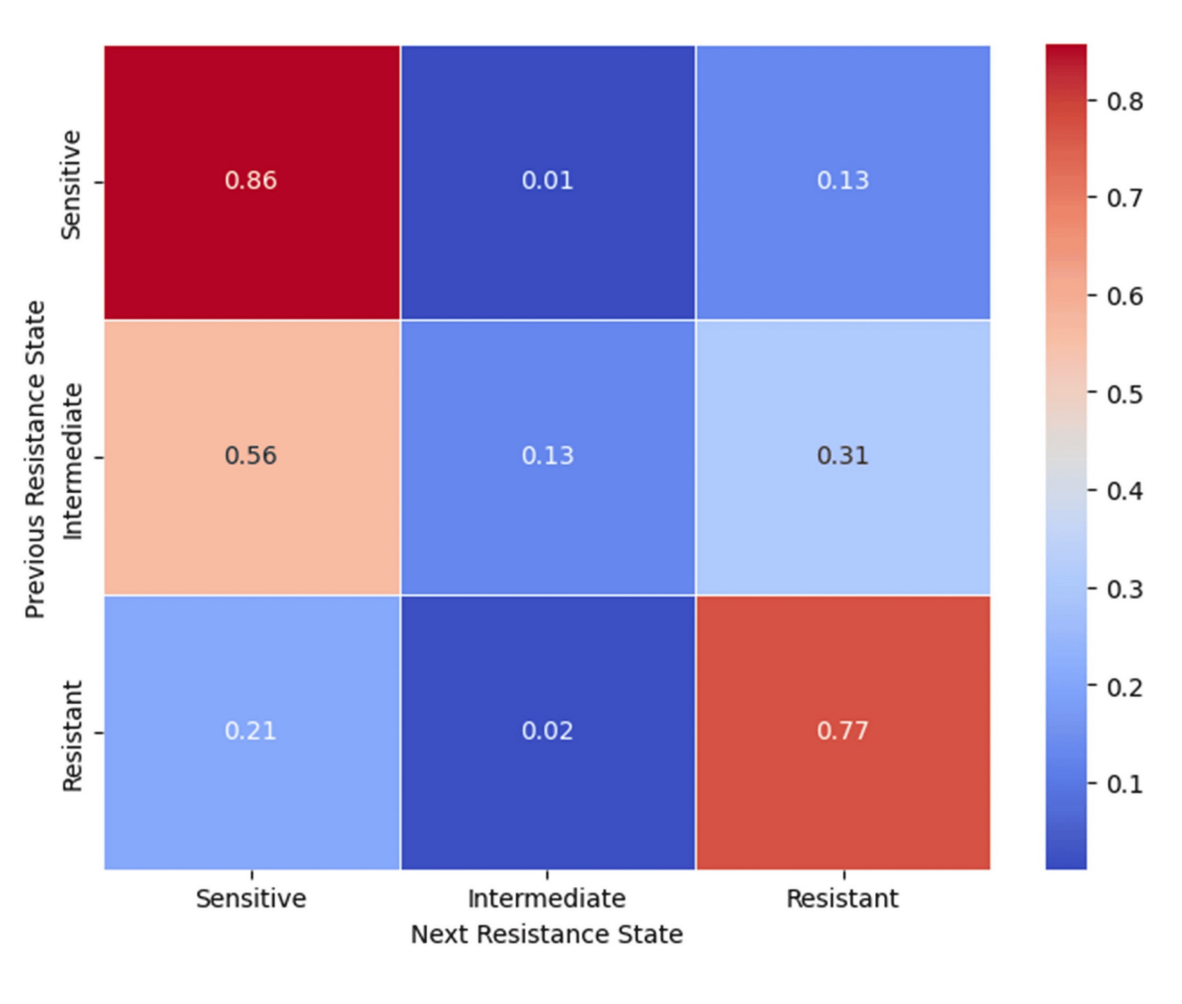
Heatmap of resistance state transitions, indicating probabilities of moving between sensitive, intermediate, and resistant states. Analysis was performed using Python v3.9.

The resistance state model (Figure 18) demonstrated that fully resistant strains persisted in that state (77%), while intermediate resistance often reverted to sensitivity (56%), and 13% of initially sensitive strains progressed to resistance.

**Figure 19:**
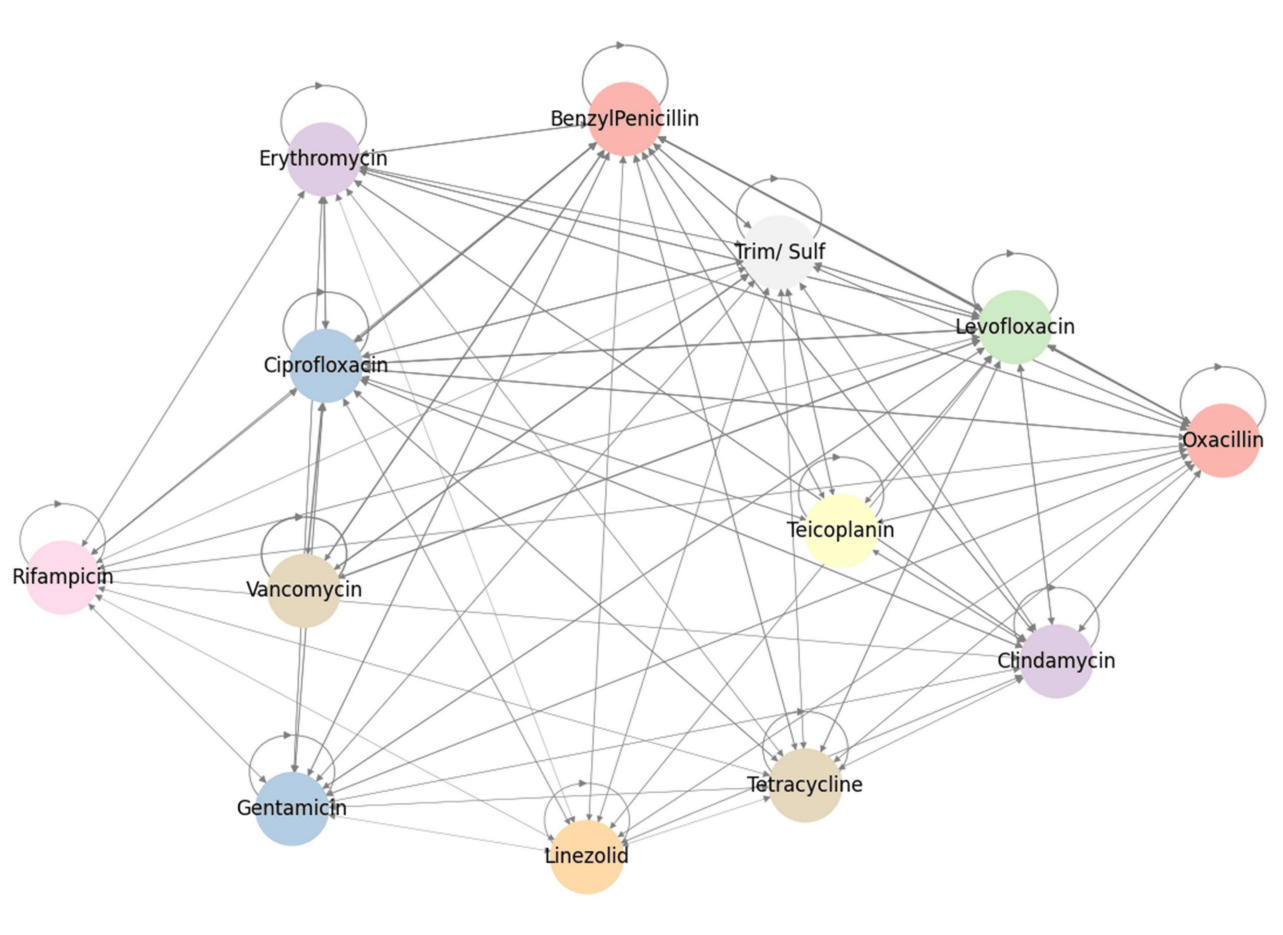
Network graph depicting the relationships and transition pathways among antibiotic resistances; node size and self-loops represent the persistence and interconnectedness of resistance traits. Analysis was performed using Python v3.9.

Network analysis (Figure 19) highlighted beta-lactams and fluoroquinolones as central hubs with stable resistance, and macrolides showed frequent transitions.

**Figure 20:**
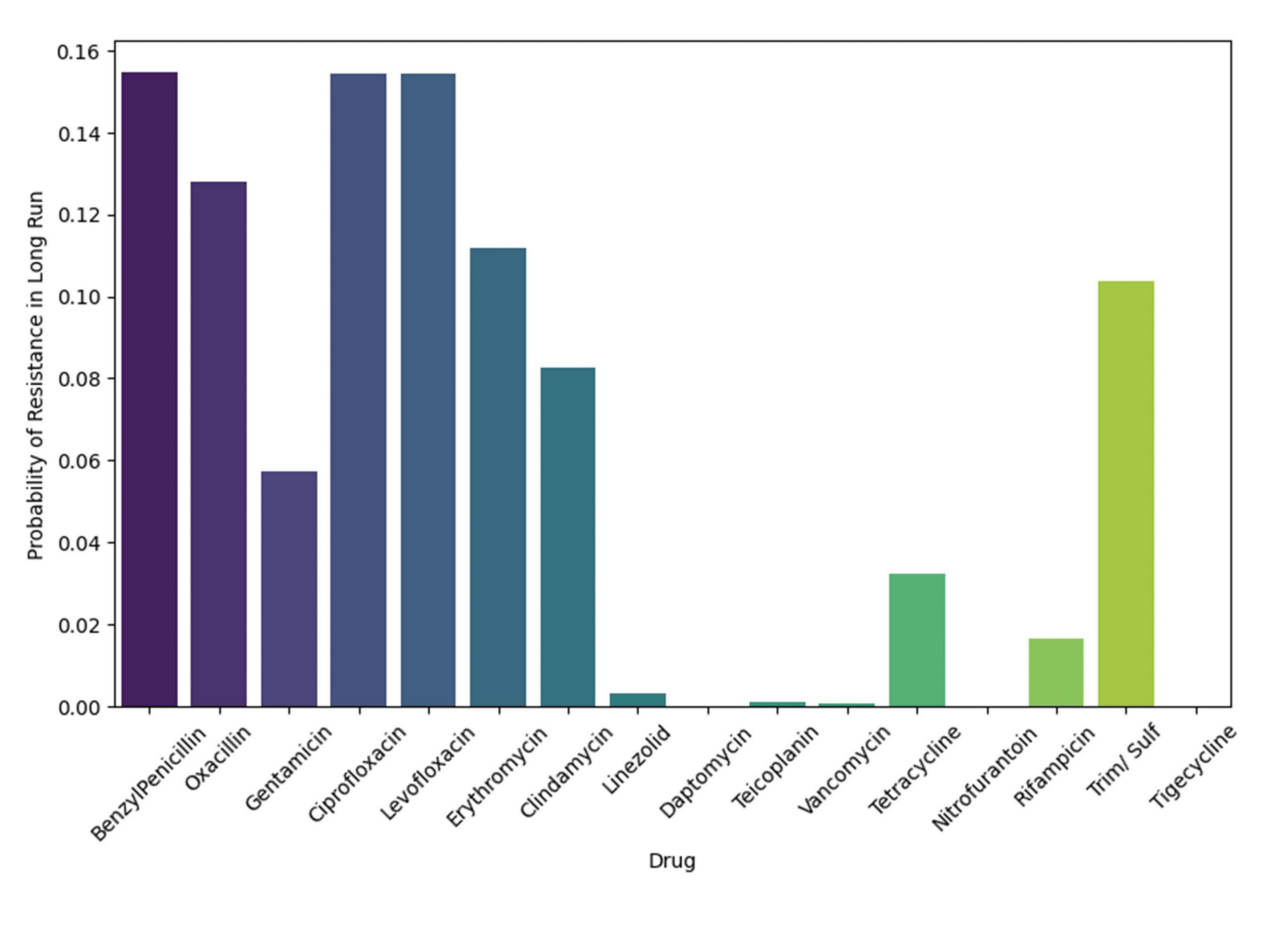
Bar plot of steady-state probabilities, reflecting the long-term likelihood of resistance persisting to each antibiotic. Analysis was performed using Python v3.9.

Finally, the steady-state probabilities (Figure 20) confirmed that resistance to beta-lactams and fluoroquinolones is persistent and widespread, whereas susceptibility to linezolid, daptomycin, and tigecycline remains largely preserved.

T7SS regulation by environmental factors

**Figure 21:**
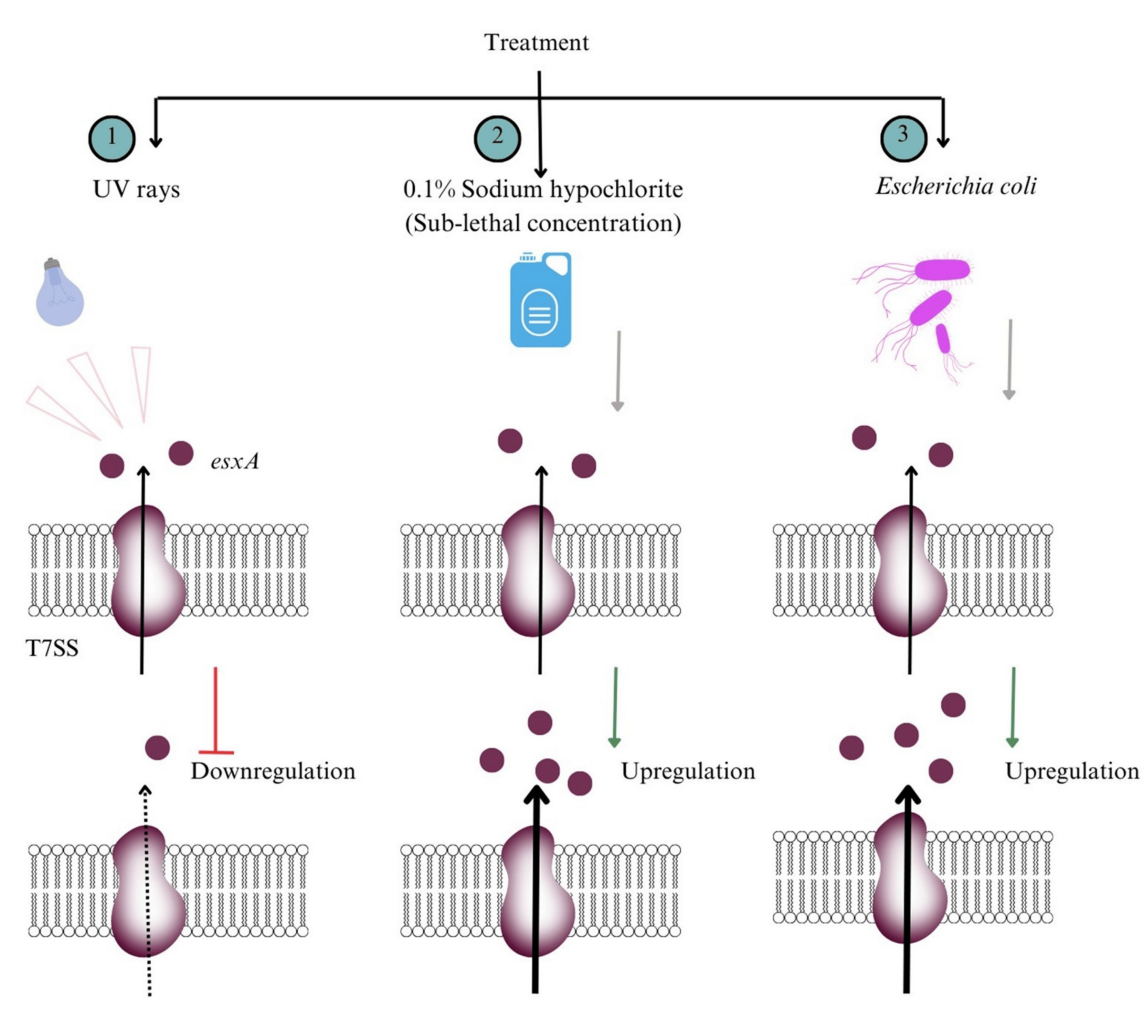
Effect of environmental treatments on T7SS expression in S. aureus. (1) UV irradiation, which led to downregulation of esxA expression and reduced T7SS activity; (2) exposure to 0.1% sodium hypochlorite, resulting in upregulation of T7SS expression; and (3) coculture with *E. coli*, which also induced upregulation of T7SS expression. Graphics were created using Canva.

To assess environmental regulation of T7SS, *S. aureus* was exposed to UV irradiation, 0.1% sodium hypochlorite, and coculture with *E. coli*. UV exposure significantly decreased T7SS expression by 1.5-fold (p < 0.05), whereas sodium hypochlorite and *E. coli* coculture increased expression by 2.5-fold and 2.2-fold, respectively (p < 0.05) (Figure 21, Appendix 3).

**Figure 22:**
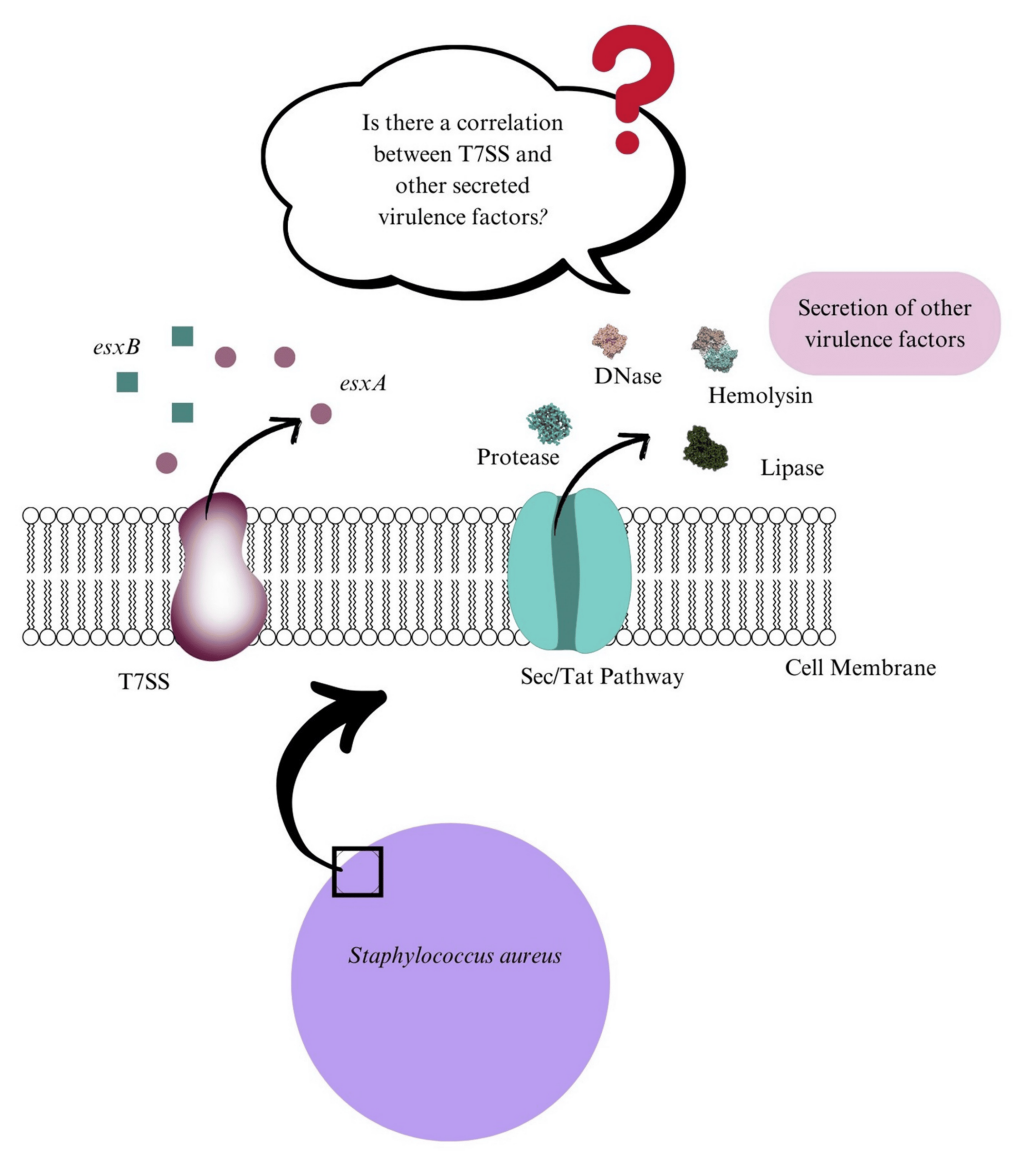
Illustration of the hypothesis: Is the T7SS correlated with other virulence factors secreted by S. aureus. Graphics were created using Canva

An illustrative conceptual model of the proposed relationship between T7SS expression and virulence determinants is presented in Figure 22.

## Discussion

All *S. aureus* isolates demonstrated capsule production, consistent with previous PCR-based findings reported by Avilés et al., suggesting that capsular polysaccharides are universally present in clinical strains [22]. The detection of the *esxA* gene in all isolates further suggests that the type VII secretion system is a conserved virulence determinant in clinical *S. aureus*. Although the gene was present in all isolates, RT-qPCR revealed variable expression levels, suggesting differential transcriptional activation of T7SS. This variability may reflect differences in regulatory control, such as modulation by global regulators (e.g., agr, SaeRS, SarA), strain-specific genetic backgrounds, quorum-sensing activity, or prior environmental exposures influencing baseline expression states. We hypothesized that T7SS expression is associated with the levels of key virulence factors, including DNase, hemolysin, protease, lipase, and staphyloxanthin (Figure 11), and our findings demonstrated significant positive correlations between T7SS expression and these determinants. The predominance of DNase and protease activity aligns with their established roles in promoting tissue invasion, degradation of neutrophil extracellular traps [23], and evasion of host immune responses [24]. In contrast, the comparatively lower production of staphyloxanthin pigment may reflect strain-specific differences in oxidative stress adaptation [25]. The presence of clear hemolytic zones confirms active hemolysin production, supporting its role in host cell membrane damage and cytotoxicity [26]. The observed positive correlations suggest that T7SS may function within a coordinated virulence program rather than as an isolated secretion pathway. Such co-expression patterns imply shared upstream regulatory circuits that synchronize extracellular enzyme production and secretion-associated effectors during infection. This integrated regulation may enhance bacterial fitness by simultaneously promoting immune evasion, tissue damage, and competitive survival within host environments. Collectively, these findings highlight the heterogeneity in virulence factor expression among clinical isolates and suggest that DNase, protease, and lipase may contribute more prominently to pathogenic potential. Although no significant correlation was observed between T7SS expression and antibiotic resistance profiles in our study, previous investigations, such as that by Rasmi et al., have reported associations between resistance characteristics and specific virulence genes, including sea and icaA, indicating that virulence-resistance interactions may be gene-specific rather than universally coordinated [27].

Principal component analysis demonstrated that EsxA expression was distributed among other virulence determinants rather than forming distinct clusters, suggesting coordinated regulation rather than independent activation. This observation aligns with the positive Pearson correlations and pairwise analyses, emphasizing the hypothesis that the T7SS component EsxA participates in the broader virulence network of *S. aureus*. Feature selection using machine learning identified protease as the most influential predictor of T7SS expression, highlighting a potential functional linkage between secretory proteolysis and T7SS activity. The unimodal and bimodal distributions observed in ridge and violin plots likely reflect heterogeneity in regulatory control, strain background, or adaptive responses to environmental pressures. Chord analysis further demonstrated strong functional coordination between EsxA and hemolysin, suggesting co-regulation between the Type VII secretion system and cytolytic virulence programs. Interestingly, the inverse association between staphyloxanthin and DNase points toward a possible biological trade-off between oxidative stress protection and extracellular nuclease activity. Finally, the Markov chain network analysis supports the concept that virulence determinants operate within an integrated regulatory framework rather than as isolated traits, potentially governed by global regulators such as SigB, SarA, SaeRS, and agr [15, 17, 28].

Methicillin resistance was observed in 74% of isolates, which, although high, is slightly lower than the 87.2% prevalence reported by Dhungel et al. [29]. The prevalence of vancomycin-resistant *S. aureus* (VRSA) in our study was low (0.6%), aligning closely with the 0% reported by Khanal et al. [30] but markedly lower than the 5.5% reported by Gwad et al., suggesting geographic variability in glycopeptide resistance patterns [31]. Notably, multidrug resistance (MDR) was detected in 88% of isolates, comparable to the 79% MDR rate reported by Alfeky et al., highlighting the persistent burden of multidrug resistance in clinical settings [32]. No extensively drug-resistant (XDR) isolates were identified in our cohort, contrasting with reports from Iran documenting a 22% XDR prevalence, which may reflect regional differences in antimicrobial stewardship and prescribing practices [33]. Furthermore, when compared with surveillance data from 2015-2017, our findings indicate emerging resistance to teicoplanin and linezolid, raising concern regarding the gradual erosion of efficacy among last-resort agents [34].

The clustering patterns observed in the co-resistance network suggest the involvement of shared resistance mechanisms, including efflux pump overexpression, plasmid-mediated gene transfer, and target-site modifications. In contrast, the relative isolation of certain antibiotics supports the presence of distinct and less interconnected resistance pathways. Co-occurrence analysis demonstrated strong cross-resistance across antibiotic classes, particularly between fluoroquinolones and β-lactams, reflecting shared molecular mechanisms and sustained selective pressure from clinical use. Markov transition modelling further indicated that resistance to vancomycin, fluoroquinolones, and benzylpenicillin may function as potential gateways toward broader multidrug resistance, whereas linezolid and tigecycline exhibited comparatively stable resistance dynamics. The resistance state model highlighted the high persistence of fully resistant phenotypes, highlighting the limited likelihood of spontaneous reversion once resistance is established. Consistently, network topology and steady-state probability analyses confirmed β-lactams and fluoroquinolones as central resistance hubs, while last-resort agents maintained largely preserved susceptibility, emphasizing their sustained therapeutic importance and the need for continued stewardship to prevent future resistance escalation.

The observed environmental modulation of T7SS expression is consistent with previous findings demonstrating stress-responsive regulation of esxA. Cincarova et al. reported increased esxA expression following chloramine T exposure, supporting our observation of upregulation under sodium hypochlorite treatment [35]. Similarly, Cao et al. demonstrated that T7SS mediates interbacterial competition through the secretion of nuclease toxins such as EsaD, which may explain the enhanced expression observed during *E. coli* coculture [36]. In contrast, the downregulation of T7SS following UV exposure parallels findings by Fila et al., who showed that light-based irradiation reduces bacterial virulence factor expression [37]. Given that many *S. aureus* virulence determinants are governed by the agr quorum-sensing system [38], our findings support the hypothesis that agr may also regulate T7SS activity. This is further substantiated by Schulthess et al., who demonstrated agr-dependent upregulation of esxA, suggesting that T7SS may be integrated within the broader agr-controlled virulence regulatory network [39].

Limitations

T7SS activity was inferred using esxA transcript levels as a representative marker; however, the Type VII secretion system comprises multiple structural and effector components, and transcription of a single gene may not fully reflect system-wide activation or secretion competence. Additionally, environmental modulation experiments assessed transcriptional responses without parallel protein-level validation. Biosynthetic or additional stress-response gene controls were not systematically evaluated when assessing environmental effects on T7SS, which may confound the interpretation of pathway-specific versus global transcriptional changes. Temporal expression dynamics across different growth phases were not examined. The study design was cross-sectional and based on isolates from a single tertiary-care center, limiting generalizability. Genetic lineage or clonal background was not characterized, which may contribute to phenotypic heterogeneity. Finally, machine-learning analyses were exploratory and conducted without external validation; therefore, identified feature importance patterns represent dataset-specific associations rather than generalizable predictive models. Additionally, since the agr system has been targeted to suppress other virulence factors [14, 40-41]. Future studies should assess whether agr inhibition also reduces T7SS expression.

## Conclusions

This study integrates wet-laboratory microbiological assays with exploratory computational analyses to examine associations between T7SS transcription and virulence phenotypes in clinical *S. aureus* isolates. Our findings demonstrate statistically significant relationships between esxA expression and several enzymatic virulence factors, and identify dataset-specific co-expression patterns through clustering and feature-ranking approaches. The machine-learning analyses were applied as exploratory tools to characterize data structure rather than to establish predictive clinical models.

Environmental modulation experiments indicate that esxA transcription is responsive to defined laboratory stress conditions, suggesting that T7SS expression may vary under different environmental contexts. However, given the transcript-level assessment and cross-sectional design, causal regulatory mechanisms cannot be inferred.

Overall, these results contribute to a structured characterization of T7SS-associated phenotypes within a clinical isolate cohort and provide a foundation for future mechanistic and protein-level investigations into T7SS regulation and its relationship with virulence expression.

## Figures and Tables

**Figure 23:**
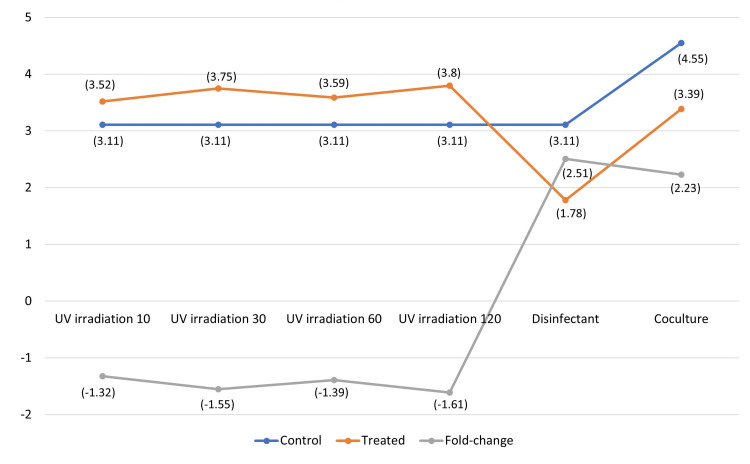
Fold change in S. aureus T7SS gene expression in response to environmental factors.

## Appendices

Appendix 1

PCR Cycle Parameters

The presence of the T7SS in clinical isolates of *S. aureus* was confirmed by polymerase chain reaction (PCR) using an Applied Biosystems Veriti 96-well thermal cycler. DNA was isolated from a 24-hour fresh culture of *S. aureus* grown on blood agar. Bacterial suspensions were prepared in nutrient broth, incubated at 37°C until log phase, and adjusted to the 0.5 McFarland standard. DNA extraction was performed using the HiPurA Bacterial Genomic DNA Purification Kit (HIMEDIA Laboratories Pvt. Ltd., Mumbai, India) according to the manufacturer’s protocol. The quality of the isolated DNA was verified by agarose gel electrophoresis using 0.8% agarose and a Tecan NanoQuant Plate™, used with the Infinite® 200 Pro microplate reader, with an absorbance ratio of 260/280. For PCR, a 25 μl reaction mixture was prepared containing 12.5 μl of Emerald dAMP GT PCR Master Mix K, 1.5 μl of template DNA, 0.8 μl of forward primer, 0.8 μl of reverse primer, 1 μl of MgCl2, and 8.4 μl of nuclease-free water. The PCR cycle parameters were as follows: initial denaturation at 94°C for 2 minutes; 30 cycles of denaturation at 94°C for 30 seconds, annealing at 54°C for 30 seconds, and extension at 72°C for 1 minute; and a final extension at 72°C for 10 minutes. The PCR products were visualised using 1.3% agarose gel electrophoresis, with 5 μl of EtBr per 100 ml of gel. A 100 bp ladder (Gene to Protein Pvt. Ltd.) was used for size comparison. For each sample, 3 μl of DNA was mixed with 3 μl of Green Track 6X Loading Dye (Gene to Protein Pvt. Ltd.) and loaded into the gel wells. Electrophoresis was conducted at 130 volts. The 16S rRNA gene of *S. aureus* was used as the positive control, and *E. coli* ATCC 25922 was used as the negative control.

Appendix 2

cDNA Synthesis and qPCR Cycle Parameters

A sample size of 84 was determined using the G*Power statistical tool for a correlation bivariate normal model test with a 95% confidence interval to achieve a power of 0.8. However, 90 clinical isolates of *S. aureus* were randomly selected for quantification of the T7SS using reverse transcriptase-quantitative polymerase chain reaction (RT-qPCR). RNA was isolated from a 24-hour fresh culture of *S. aureus* grown on blood agar. Bacterial suspensions were prepared in nutrient broth, incubated at 37°C until log phase, and adjusted to the 0.5 McFarland standard. RNA extraction was performed using the HiPurA Bacterial RNA Purification Kit (HIMEDIA Laboratories Pvt. Ltd., Mumbai, India) according to the manufacturer’s protocol. The purity and quantity of the isolated RNA were measured using a Tecan NanoQuant Plate™ with the Infinite® 200 Pro microplate reader, using an absorbance ratio of 260/280. The RNA was reverse transcribed into cDNA using a synthesis kit from G-Biosciences (St. Louis, MO, USA) following the manufacturer's instructions. The RT reaction mixture (10 μl) consisted of 5 μl of 2X RT Easy Mix, 0.5 μl of random primer, 0.5 μl of oligo (dT) primer, total RNA (<2.5 μg), and nuclease-free water to a total volume of 10 μl. The program for reverse transcription was set to 42°C for 20 minutes, followed by inactivation at 85°C for five minutes.

The primers used for the esxA gene are listed in Table 1. Quantitative PCR was performed using SYBR™ Green qPCR Master Mix following the manufacturer’s protocol. The master mix (20 μl) included 10 μl of 2X AB HS SYBR Green qPCR Mix, 2 μl of forward primer, 2 μl of reverse primer, 2 μl of cDNA template, and 4 μl of double-distilled water. qPCR was performed using a C1000 Touch™ Thermal Cycler with a CFX96™ Real-Time PCR Detection System, with a program comprising an initial denaturation at 94°C for two minutes, followed by 35 cycles of 94°C for 30 seconds (denaturation), 54°C for 30 seconds (annealing), and 72°C for one minute. The 16S rRNA gene of *S. aureus* was used as an internal positive control (primer sequences in Table 1), and a nontemplate control served as the negative control. The qPCR products were visualized using 1.3% agarose gel electrophoresis to verify the specificity of the band size (198 bp).

Appendix 3

## References

[1] Cheung GY, Bae JS, Otto M (2021). Pathogenicity and virulence of Staphylococcus aureus. Virulence.

[2] Ezeh CK, Eze CN, Dibua ME, Emencheta SC (2023). A meta-analysis on the prevalence of resistance of Staphylococcus aureus to different antibiotics in Nigeria. Antimicrob Resist Infect Control.

[3] Yehia FAA, Yousef N, Askoura M (2021). Exploring Staphylococcus aureus virulence factors; special emphasis on staphyloxanthin. Microbiology and Biotechnology Letters.

[4] Bear A, Locke T, Rowland-Jones S, Pecetta S, Bagnoli F, Darton TC (2023). The immune evasion roles of Staphylococcus aureus protein A and impact on vaccine development. Front Cell Infect Microbiol.

[5] Pelz A, Wieland KP, Putzbach K, Hentschel P, Albert K, Götz F (2005). Structure and biosynthesis of staphyloxanthin from Staphylococcus aureus. J Biol Chem.

[6] Sharaf MH, El-Sherbiny GM, Moghannem SA, Abdelmonem M, Elsehemy IA, Metwaly AM, Kalaba MH (2021). New combination approaches to combat methicillin-resistant Staphylococcus aureus (MRSA). Sci Rep.

[7] Hassoun A, Linden PK, Friedman B (2017). Incidence, prevalence, and management of MRSA bacteremia across patient populations-a review of recent developments in MRSA management and treatment. Crit Care.

[8] Bowman L, Palmer T (2021). The type VII secretion system of Staphylococcus. Annu Rev Microbiol.

[9] Burts ML, Williams WA, DeBord K, Missiakas DM (2005). EsxA and EsxB are secreted by an ESAT-6-like system that is required for the pathogenesis of Staphylococcus aureus infections. Proc Natl Acad Sci U S A.

[10] Dai Y, Wang Y, Liu Q (2017). A novel ESAT-6 secretion system-secreted protein EsxX of community-associated Staphylococcus aureus Lineage ST398 contributes to immune evasion and virulence. Front Microbiol.

[11] Kengmo Tchoupa A, Watkins KE, Jones RA (2020). The type VII secretion system protects Staphylococcus aureus against antimicrobial host fatty acids. Sci Rep.

[12] Garrett SR, Palmer T (2024). The role of proteinaceous toxins secreted by Staphylococcus aureus in interbacterial competition. FEMS Microbes.

[13] Cruciani M, Etna MP, Camilli R (2017). Staphylococcus aureus Esx factors control human dendritic cell functions conditioning Th1/Th17 response. Front Cell Infect Microbiol.

[14] Bezar IF, Mashruwala AA, Boyd JM, Stock AM (2019). Drug-like fragments inhibit agr-mediated virulence expression in Staphylococcus aureus. Sci Rep.

[15] Liu X, Wang Y, Chang W, Dai Y, Ma X (2024). AgrA directly binds to the promoter of vraSR and downregulates its expression in Staphylococcus aureus. Antimicrob Agents Chemother.

[16] Jenul C, Horswill AR (2019). Regulation of Staphylococcus aureus virulence. Microbiol Spectr.

[17] Wittekind MA, Briaud P, Smith JL, Tennant JR, Carroll RK (2023). The small protein ScrA influences Staphylococcus aureus virulence-related processes via the SaeRS system. Microbiol Spectr.

[18] Nirmala B, Pai MO, Badoni G, Verma GK (2025). Machine learning analysis of type VII secretion system expression and its relationships with virulence traits and antibiotic resistance in Staphylococcus aureus. Research Square.

[19] (2026). Basic Local Alignment Search Tool. https://blast.ncbi.nlm.nih.gov/Blast.cgi.

[20] Bikandi J, San Millán R, Rementeria A, Garaizar J (2004). In silico analysis of complete bacterial genomes: PCR, AFLP-PCR and endonuclease restriction. Bioinformatics.

[21] Nirmala B, Manhas PL, Jadli M, Sharma R, Manhas H, Omar BJ (2024). A novel dual-staining method for cost-effective visualization and differentiation of microbial biofilms. Sci Rep.

[22] Echániz-Aviles G, Velazquez-Meza ME, Rodríguez-Arvizu B, Carnalla-Barajas MN, Noguerón AS (2022). Detection of capsular genotypes of methicillin-resistant Staphylococcus aureus and clonal distribution of the cap5 and cap8 genes in clinical isolates. Arch Microbiol.

[23] Sharma P, Garg N, Sharma A, Capalash N, Singh R (2019). Nucleases of bacterial pathogens as virulence factors, therapeutic targets and diagnostic markers. Int J Med Microbiol.

[24] Pietrocola G, Nobile G, Rindi S, Speziale P (2017). Staphylococcus aureus manipulates innate immunity through own and host-expressed proteases. Front Cell Infect Microbiol.

[25] Múnera-Jaramillo J, López GD, Suesca E, Carazzone C, Leidy C, Manrique-Moreno M (2024). The role of staphyloxanthin in the regulation of membrane biophysical properties in Staphylococcus aureus. Biochim Biophys Acta Biomembr.

[26] Divyakolu S, Chikkala R, Ratnakar KS (2019). Hemolysins of Staphylococcus aureus—an update on their biology, role in pathogenesis and as targets for anti-virulence therapy. Adv Infect Dis vol. 09, no.

[27] Rasmi AH, Ahmed EF, Darwish AM, Gad GF (2022). Virulence genes distributed among Staphylococcus aureus causing wound infections and their correlation to antibiotic resistance. BMC Infect Dis.

[28] Wen Z, Chen C, Shang Y (2024). Baohuoside I inhibits virulence of multidrug-resistant Staphylococcus aureus by targeting the transcription Staphylococcus accessory regulator factor SarZ. Phytomedicine.

[29] Dhungel S, Rijal KR, Yadav B (2021). Methicillin-resistant Staphylococcus aureus (MRSA): prevalence, antimicrobial susceptibility pattern, and detection of mecA gene among cardiac patients from a tertiary care heart center in Kathmandu, Nepal. Infect Dis (Auckl).

[30] Khanal LK, Sah AK, Adhikari RP, Khadka S, Sapkota J, Rai SK (2023). Prevalence and molecular characterization of methicillin resistant Staphylococcus aureus (MRSA) and vancomycin resistant Staphylococcus aureus (VRSA) in a tertiary care hospital. Nepal Medical College Journal.

[31] A. Nagy, I. A. Gwad, K. A. Al-Ghareeb, M. Ashrafbadr, G. El Din, and A. Fargazmy (28). Prevalence of vancomycin-resistant Staphylococcus aureus (VRSA) in some Egyptian hospitals.

[32] Alfeky AE, Tawfick MM, Ashour MS, El-Moghazy AA (2022). High prevalence of multi-drug resistant methicillin-resistant Staphylococcus aureus in tertiary Egyptian hospitals. J Infect Dev Ctries.

[33] Moosavian M, Baratian Dehkordi P, Hashemzadeh M (2020). Characterization of SCCmec, spa types and multidrug resistant of methicillin-resistant Staphylococcus aureus isolates in Ahvaz, Iran. Infect Drug Resist.

[34] Kot B, Wierzchowska K, Piechota M, Grużewska A (2020). Antimicrobial resistance patterns in methicillin-resistant Staphylococcus aureus from patients hospitalized during 2015-2017 in hospitals in Poland. Med Princ Pract.

[35] Cincarova L, Polansky O, Babak V, Kulich P, Kralik P (2016). Changes in the expression of biofilm-associated surface proteins in Staphylococcus aureus food-environmental isolates subjected to sublethal soncentrations of disinfectants. Biomed Res Int.

[36] Cao Z, Casabona MG, Kneuper H, Chalmers JD, Palmer T (2016). The type VII secretion system of Staphylococcus aureus secretes a nuclease toxin that targets competitor bacteria. Nat Microbiol.

[37] Fila G, Kawiak A, Grinholc MS (2017). Blue light treatment of Pseudomonas aeruginosa: strong bactericidal activity, synergism with antibiotics and inactivation of virulence factors. Virulence.

[38] Mahdally NH, George RF, Kashef MT, Al-Ghobashy M, Murad FE, Attia AS (2021). Staquorsin: a novel Staphylococcus aureus agr-mediated quorum sensing inhibitor impairing virulence in vivo without notable resistance development. Front Microbiol.

[39] Schulthess B, Bloes DA, Berger-Bächi B (2012). Opposing roles of σB and σB-controlled SpoVG in the global regulation of esxA in Staphylococcus aureus. BMC Microbiol.

[40] Sully EK, Malachowa N, Elmore BO (2014). Selective chemical inhibition of agr quorum sensing in Staphylococcus aureus promotes host defense with minimal impact on resistance. PLoS Pathog.

[41] Salam AM, Quave CL (2018). Targeting virulence in Staphylococcus aureus by chemical inhibition of the accessory gene regulator system in vivo. mSphere.

